# Knowledge, attitude, and perception of exercises among post-hematopoietic stem cell transplant patients: A cross-sectional study

**DOI:** 10.1097/MD.0000000000040036

**Published:** 2024-11-08

**Authors:** Na Han, Changqing Wu, Na Liu, Yu Deng, Li Zhang, Yan Zhu

**Affiliations:** a Department of Hematology, Zhujiang Hospital, Southern Medical University, Guangzhou, Guangdong, China.

**Keywords:** attitude, cross-sectional study, exercise tolerance, hematopoietic stem cell transplantation, knowledge, practice

## Abstract

Exercise rehabilitation is crucial for the recovery after hematopoietic stem cell transplantation (HSCT). This study aimed to investigate knowledge, attitude, and perception (KAP) of exercise among post-HSCT patients. This cross-sectional study was conducted at Zhujiang Hospital, Southern Medical University between January 2020 and December 2022 among post-HSCT patients, using a self-designed questionnaire. A total of 192 patients were included, with the mean age of 37.90 ± 11.96 years; 116 (60.42%) reported to exercise before. The mean KAP scores were 9.22 ± 2.05 (possible range: 0–12), 43.51 ± 5.47 (possible range: 12–60) and 51.79 ± 7.45 (possible range: 15–75), respectively. Patients previously inactive in exercise exhibited significant differences in KAP scores from active patients: attitude total score, positive attitude, perception total score, willingness to exercise, aerobic exercise, breathing training (all *P* < .001), with a noteworthy distinction in resistance exercise (*P* = .018). According to structural equation modeling, perception was directly influenced by knowledge (β = 0.87, *P* < .001), attitude (β = 0.26, *P* = .007), and exercise habits (β = 3.36, *P* = .001), as well as indirectly by education (β = 0.44, *P* = .010) and knowledge (β = 0.18, *P* = .029). Post-HSCT patients had adequate knowledge, moderate attitude and perception of exercises, even 1 year after HSCT. Patients inactive in exercises exhibited significant differences in knowledge and exercise perception from active patients. Healthcare professionals should tailor education, target attitude, and personalize exercise plans to facilitate effective recovery post-HSCT.

## 1. Introduction

Hematopoietic stem cell transplantation (HSCT) is the infusion of autologous or allogeneic stem cells with the goal of reestablishing hematopoietic function.^[1]^ Almost 99% of autologous and 88% of allogeneic HSCTs are performed for hematological malignancies, and rest for benign conditions, such as bone marrow failure or autoimmune diseases.^[2,3]^ With the demand for HSCT on the rise, more than 50,000 procedures are performed annually worldwide,^[4]^ and with that the prevalence of posttransplant patients is expected to exceed 500,000 in the next ten years.^[5]^ Although transplantation techniques recently advanced enough to decrease treatment-related mortality, posttransplant complications still commonly occur in HSCT recipients, making supportive care the research priority.^[1,5]^

In addition to the general fatigue caused by pretransplantation chemotherapy and posttransplant complications, isolation and prolonged bed rest after the HSCT procedure notably limit physical activity, causing muscle weakness, as well as decline in physical function, mental function and quality of life.^[6,7]^ Moreover, physical activity and exercise tolerance after HSCT is the predictive marker for later social reintegration.^[8]^ Thus, additional exercise programs based on the cardiac rehabilitation are discussed in the context of improving posttransplant well-being in combination with medical treatment.^[9,10]^ Previous studies reported a beneficial effect or exercises on psychoemotional health and quality of life, without notable adverse events.^[6,11–13]^ At the same time, partaking in the exercise-based rehabilitation put additional pressure on patient, while prevention and control of transplant complications as well as effect on hematologic malignancy itself is still under discussion.^[14]^

Recent studies showed that educational interventions aimed to improve knowledge of side effects, risks and complications of HSCT could reduce anxiety and depression, improving patients’ engagement and compliance.^[9,10]^ Knowledge, attitude and practice study is a specialized survey method that allows to access the current biopsychosocial factors associated with treatment compliance.^[15]^ Majority of current studies discussing knowledge or attitude in HSCT include medical students or professionals and demonstrate promising knowledge and understanding of HSCT rehabilitation.^[16–18]^ However, in the studies undertaken among patients who are planning to undergo HSCT or posttransplant survivors’ noncompliance is reported often.^[15]^ While exercise-based rehabilitation has been recognized as a crucial intervention for successful recovery in HSCT patients, there is a lack of comprehensive understanding regarding patients’ knowledge, attitude, and perception (KAP) of exercises. To the best of our knowledge only 1 small case study was published before^[19]^ and larger study might provide valuable insights to healthcare professionals, enabling them to better understand patients’ needs and concerns, and consequently deliver personalized medical interventions and education to enhance treatment compliance and prevent complications.

This study aims to analyze KAPs of post-HSCT patients towards exercising. Additionally, it was hypothesized that duration after the HSCT procedure and previous exercise habit would influence the KAP scores of patients; differences in the attitude between patients who partook in exercise before and those who don’t might help to uncover new barriers and facilitators for better practice of rehabilitation exercises.

## 2. Methods

### 2.1. Study design and patients

This cross-sectional study was conducted at Zhujiang Hospital, Southern Medical University between January 2020 and December 2022. Post-HSCT patients aged between 18 and 65 years were included. Patients with severe complications, cognitive impairments, or those unable to cooperate due to mental illness were excluded. This study was approved by the Ethics Committee of Zhujiang Hospital, Southern Medical University (Ethical approval number: 2023-KY-190-02), and informed consent was obtained from patients before completing the questionnaire.

### 2.2. Procedures

The questionnaire comprised 4 dimensions and was developed following the Guidelines for Exercise and Cancer published by American college of sports medicine,^[20]^ Clinical Practice Guidelines in Oncology for Hematopoietic Cell Transplantation,^[1]^ and previous studies addressing exercise training programs for patients undergoing HSCT.^[19,21]^ Subsequently, the initial questionnaire design underwent revisions based on feedback from 3 hematology experts. Redundant or repetitive questions were eliminated, and certain statements were refined and clarified through adjustments made in response to their valuable insights. The final questionnaire, distributing in Chinese (a version translated into English was attached as a Table 1), included the following dimensions: (1) Demographic characteristics include 10 items. (2) Knowledge dimension consists of 12 items. One point was awarded for a correct answer, while an incorrect or unclear answer received 0 points. (3) Attitude dimension includes 12 items. A 5-point Likert scale was used, ranging from “very positive” (5 points) to “very negative” (1 point). (4) Perception dimension involves 15 items, with the same 5-point Likert scale was also used, ranging from “always” (5 points) to “never” (1 point). A final score exceeding 75% of the total score indicates an adequate level of knowledge, positive attitude, and perception.^[22]^ A score ranging from 50% to 75% of the total score indicates a moderate level of KAP. On the other hand, a score below 50% of the total score signifies inadequate knowledge, negative attitude, and perception.

**Table 1:** Surgery ID.

Dear Patient, We are researchers from the Zhujiang Hospital of Southern Medical University, and we cordially invite you to participate in our research project. The purpose of this study is to understand the knowledge, attitudes, and practices toward exercise among patients who have undergone hematopoietic stem cell transplantation. The findings aim to provide a basis for the development of scientifically informed intervention strategies, potentially benefiting a larger population and improving their health outcomes in the future. Your participation in this study is voluntary, and ethical approval has been obtained from the review committee. If you agree to participate, please refer to the following instructions: 1. Kindly complete the questionnaire; there are no right or wrong answers. Simply provide information based on your actual experiences. Feel free to reach out to us with any questions during the answering process, and please submit the completed questionnaire in a timely manner. 2. This study involves a straightforward questionnaire survey that will not cause harm to your physical or mental well-being. However, it will touch upon some private information, such as your gender and age. Be assured that we will strictly maintain confidentiality and not disclose your information. Please feel confident in filling out the questionnaire. 3. As a participant, you can inquire about information related to this study and its progress at any time. If you decide to withdraw from the study, please inform us, and your data will not be included in the research results. Finally, we sincerely appreciate your valuable time and support for our scientific research! □ I am aware of and consent to the use of the collected data for scientific research. Informed Consent Signature: Participation Date: Year Month Day					
Part 1 Basic information					
1. Gender	a. Male b. Female				
2. Age					
3. Residence	a. Rural b. Urban				
4. Education	a. Elementary school and below b. Junior high school c. High school/technical school d. College/bachelor’s degree e. Graduate and above				
5. Employment status	a. Employed b. Unemployed				
6. In the past year, the average monthly per capita income in your household (including in-kind income and rental income, etc):______CNY	a. <2000 b. 2000–5000 c. 5000–10,000 d. –20,000 e. >20,000				
7. Transplantation Date: Year Month					
8. Diagnosis	a. Acute lymphocytic leukemia b. Chronic myeloid leukemia c. Lymphoma d. Multiple myeloma e. Other				
9. Type of transplantation	a. Autologous hematopoietic stem cell transplantation b. Allogeneic hematopoietic stem cell transplantation				
10. Previous exercise habits	a. Yes b. No				
Part 2 Understanding of exercise					
1. The standard treatment for hematopoietic stem cell transplantation is to isolate patients in a laminar flow ward for about 1 month, during which safe and effective exercise should be performed to develop exercise habits.			a. Yes	b. No	c. Uncertain
2. Exercise can improve muscle atrophy and joint stiffness, increase muscle strength, improve cardiovascular function, and alleviate fatigue			a. Yes	b. No	c. Uncertain
3. Taking corticosteroid drugs (such as prednisone) may lead to muscle weakness, muscle atrophy, and osteoporosis, therefore patients taking steroids should avoid exercise (wrong)			a. Yes	B. No	c. Uncertain
4. Exercise helps increase appetite, improve sleep, regulate emotions, and reduce depression and anxiety			a. Yes	b. No	c. Uncertain
5. During hospitalization, bed rest is required, and high-intensity activities should be avoided, but it does not mean no exercise at all (wrong)			a. Yes	b. No	c. Uncertain
6. Muscle strength is crucial because it is closely related to independence in daily activities			a. Yes	b. No	c. Uncertain
7. Muscle atrophy (muscle wasting) is one of the severe complications in hematopoietic stem cell transplant patients, affecting rehabilitation and prognosis			a. Yes	b. No	c. Uncertain
8. Patients’ low physical activity leads to a decrease in skeletal muscle mass, resulting in skeletal muscle atrophy, and muscle loss			a. Yes	B. No	c. Uncertain
9. After transplantation, patients can engage in exercise if their physical condition permits, but they should proceed with caution and gradually increase the intensity			a. Yes	b. No	c. Uncertain
10. The principle of posttransplant exercise is to avoid exhaustion, starting from indoor activities, and gradually increasing the intensity			a. Yes	b. No	c. Uncertain
11. Posttransplant activities can include walking, yoga, tai chi, and simple stretching exercises, and preferably during less crowded times			a. Yes	b. No	c. Uncertain
12. To ensure safety, patients should engage in exercise when their platelet count is >10 × 10^9^/L, absolute neutrophil count is >0.5 × 10^9^/L, hemoglobin level is >80 g/L, and there are no severe infections or bleeding episodes, and no pathological fractures			a. Yes	b. No	c. Uncertain
Part 3 Attitudes and thoughts towards exercise after transplantation					
For the following statements about exercise after transplantation, please indicate your viewpoint:					
1. During the transplantation period, the discomfort in my body makes me extremely miserable, and I have no desire to exercise at all (N)	a. strongly agree	b. agree	c. neutral	d. disagree	e. strongly disagree
2. The discomfort during the transplantation period makes it difficult for me to intake nutrition, therefore, I am unable to exercise (N)	a. Strongly agree	b. Agree	c. Neutral	d. Disagree	e. Strongly disagree
3. Even if I know I can exercise, I have no desire to do anything during the transplantation period (N)	a. Strongly agree	b. Agree	c. Neutral	d. Disagree	e. Strongly disagree
4. Being confined to the ward makes me anxious, and I have no intention to exercise at all (N)	a. Strongly agree	b. Agree	c. Neutral	d. Disagree	e. Strongly disagree
5. If I hear that other patients also don’t want to exercise, I will be influenced and become resistant to exercise (N)	a. Strongly agree	b. Agree	c. Neutral	d. Disagree	e. Strongly disagree
If the following situations arise, would you be more willing to exercise and persevere?					
6. The doctor talked to me about the benefits of exercise and advised me to exercise (P)	a. Strongly agree	b. Agree	c. Neutral	d. Disagree	e. Strongly disagree
7. The exercise regimen is relatively simple and easy (P)	a. Strongly agree	b. Agree	c. Neutral	d. Disagree	e. Strongly disagree
8. There is guidance, supervision, and encouragement from professionals in the field of exercise (P)	a. Strongly agree	b. Agree	c. Neutral	d. Disagree	e. Strongly disagree
9. Hearing other patients describe the benefits of exercise (P)	a. Strongly agree	b. Agree	c. Neutral	d. Disagree	e. Strongly disagree
Statements you agree with:					
10. Willpower is important, as it motivates me to continue exercise even if I feel physically uncomfortable, with the determination to resume after recovery (P)	a. Strongly agree	b. Agree	c. Neutral	d. Disagree	e. Strongly disagree
11. I focus on positive thoughts that exercise will make me better, so I will exercise actively (P)	a. Strongly agree	b. Agree	c. Neutral	d. Disagree	e. Strongly disagree
12. Exercise enables me to achieve my goals, so I will exercise actively (P)	a. Strongly agree	b. Agree	c. Neutral	d. Disagree	e. Strongly disagree
Part 4 Behavioral practices towards exercise					
1. The frequency with which you consider the following factors during the exercise process (options are arranged in descending order of frequency):					
1.1 Quality of air (P)	a. Always	b. Often	c. Sometimes	d. Rarely	e. Never
1.2 Availability of family members for accompanying (P)	a. Always	b. Often	c. Sometimes	d. Rarely	e. Never
1.3 Weather changes and adjustments in clothing (P)	a. Always	b. Often	c. Sometimes	d. Rarely	e. Never
1.4 Blood indicators (P)	a. Always	b. Often	c. Sometimes	d. Rarely	e. Never
1.5 Presence of healthcare personnel accompanying (P)	a. Always	b. Often	c. Sometimes	d. Rarely	e. Never
Selection of willingness for the following types of exercises:					
2. Aerobic exercises:					
2.1 Walking (P)	a. Very willing	b. Willing	c. Neutral	d. Unwilling	e. Very unwilling
2.2 Jogging/Running (P)	a. Very willing	b. Willing	c. Neutral	d. Unwilling	e. Very unwilling
2.3 Climbing stairs (P)	a. Very willing	b. Willing	c. Neutral	d. Unwilling	e. Very unwilling
2.4 Cycling (P)	a. Very willing	b. Willing	c. Neutral	d. Unwilling	e. Very unwilling
3. Resistance exercises:					
3.1 Bodyweight exercises (e.g., sit-ups, squats) (P)	a. Very willing	b. Willing	c. Neutral	d. Unwilling	e. Very unwilling
3.2 Resistance bands (P)	a. Very willing	b. Willing	c. Neutral	d. Unwilling	e. Very unwilling
3.3 Dumbbells (P)	a. Very willing	b. Willing	c. Neutral	d. Unwilling	e. Very unwilling
3.4 Weight training equipment (common gym equipment such as Smith machine, leg press machine): (P)	a. Very willing	b. Willing	c. Neutral	d. Unwilling	e. Very unwilling
4. Breathing exercises:					
4.1 Breathing technique training (e.g., pursed lip breathing, abdominal breathing) (P)	a. Very willing	b. Willing	c. Neutral	d. Unwilling	e. Very unwilling
4.2 Respiratory muscle training (e.g., resistance breathing, breathing exercises) (P)	a. Very willing	b. Willing	c. Neutral	d. Unwilling	e. Very unwilling

A small-scale pilot study involving 34 patients was conducted to test reliability. The internal consistency of the questionnaire was evaluated high internal consistency with Cronbach *α* was 0.916. To confirm the study patients were post-HSCT patients, 2 recruitment methods were used. The first involved inviting patients during their clinic follow-up visits at a single medical center. The second method utilized the hospital’s patient follow-up system for data collection during telephone follow-ups. To ensure the study’s success, the research assistant team underwent specialized training and received detailed guidance materials. They were trained in research procedures, data collection methods, and ethical guidelines. During the questionnaire completion, research assistants offered oral explanations to patients’ questions without influencing their answers. An online questionnaire was designed using a social media-based Wen Juan Xing platform (https://www.wjx.cn) and a quick response code was generated for data collection via WeChat messenger. Patients scanned the quick response code to log in and complete the questionnaire. The questionnaires were anonymous. To ensure the quality and completeness of the questionnaire results, each IP address was restricted to 1 submission, and all items were mandatory and anonymous. The research team members checked the completeness, internal coherence, and reasonableness of all questionnaires.

### 2.3. Statistical analysis

The statistical analysis software used was Stata 17.0 (Stata Corporation, College Station, TX). Continuous variables were described using mean ± standard deviation, and group comparisons were conducted using *t* tests or analysis of variance. Categorical variables were described using n (%). The correlation between KAP scores was assessed using Pearson correlation analysis. Structural equation modeling (SEM) was employed to assess the hypotheses: (1) Education directly affects knowledge and attitude; (2) Knowledge directly influences attitude and perception; (3) Exercise habits have a direct impact on attitude and perception; and (4) Attitude directly affects perception. A 2-sided *P* < .05 was considered significantly different.

## 3. Results

A total of 200 questionnaires were distributed, and after excluding 8 cases, finally, 192 valid questionnaires remained, with an effective response rate of 96.0%. The mean age of the patients was 37.90 ± 11.96 years, and 85 (44.97%) underwent transplantation within 12 months, while 104 (55.03%) had the procedure after 12 months. The initial diagnosis was acute lymphocytic leukemia for 87 (45.31%) patients and other malignancies for 105 (54.69%) patients. Among them, 154 (80.21%) received allogeneic HSCT, while 38 (19.79%) received autologous HSCT. Regarding exercise habits, 116 (60.42%) of the patients reported having exercised previously, while 76 (39.58%) had not participated in exercise-based rehabilitation before (Table 2). The mean KAP scores were 9.22 ± 2.05 (possible range: 0–12), 43.51 ± 5.47 (possible range: 12–60) and 51.79 ± 7.45 (possible range: 15–75), respectively. Further details regarding the distribution of scores across questions can be observed in Table 3.

**Table 2:** Demographic characteristics.

Variables	N (%)	Knowledge score		Attitude score		Perception score	
		Mean ± SD	*P*	Mean ± SD	*P*	Mean ± SD	*P*
Total	192	9.22 ± 2.05		43.51 ± 5.47		51.79 ± 7.45	
Gender			.674		.205		.740
Female	105 (54.69)	9.16 ± 2.25		43.96 ± 5.75		51.63 ± 7.34	
Male	87 (45.31)	9.29 ± 1.78		42.95 ± 5.09		51.99 ± 7.62	
Age	37.90 ± 11.96						
Residence			.086		.322		.798
Urban	90 (46.88)	9.49 ± 1.95		43.92 ± 5.59		51.64 ± 7.50	
Rural	102 (53.12)	8.98 ± 2.11		43.14 ± 5.36		51.92 ± 7.44	
Education			.003		.096		.005
High school/vocational	46 (23.96)	9.00 ± 2.44		42.02 ± 5.14		50.26 ± 8.03	
Junior high school and below	74 (38.54)	8.74 ± 1.92		43.76 ± 5.01		50.55 ± 6.43	
College and above	72 (37.50)	9.85 ± 1.74		44.19 ± 6.00		54.04 ± 7.60	
Employment status			.342		.981		.025
Ref employed	44 (22.92)	9.48 ± 1.77		43.52 ± 6.22		54.00 ± 7.86	
Unemployed	148 (77.08)	9.14 ± 2.12		43.50 ± 5.25		51.14 ± 7.22	
Monthly per capita income, CNY			.124		.251		.222
2000–5000	50 (26.04)	9.10 ± 2.12		42.50 ± 5.15		49.90 ± 6.80	
<2000	55 (28.65)	8.76 ± 2.32		43.80 ± 4.98		52.31 ± 8.43	
5000–10,000	53 (27.60)	9.66 ± 1.52		43.26 ± 5.88		52.62 ± 7.30	
>10,000	34 (17.71)	9.44 ± 2.11		44.88 ± 5.91		52.44 ± 6.72	
Duration of transplantation (months)			.076		.868		.853
≤12 months	85 (44.97)	9.49 ± 1.64		43.58 ± 5.39		51.79 ± 7.36	
>12 months	104 (55.03)	8.96 ± 2.32		43.44 ± 5.62		51.59 ± 7.51	
Diagnosis			.623		.617		.243
Acute lymphocytic leukemia	87 (45.31)	9.30 ± 2.06		43.29 ± 5.09		52.48 ± 7.67	
Other	105 (54.69)	9.15 ± 2.05		43.69 ± 5.79		51.22 ± 7.25	
Transplant type			.393		.167		.183
Allogeneic hematopoietic stem cell transplant	154 (80.21)	9.16 ± 2.15		43.23 ± 5.21		51.44 ± 7.24	
Autologous hematopoietic stem cell transplant	38 (19.79)	9.47 ± 1.59		44.61 ± 6.38		53.24 ± 8.22	
Previous exercise habits			.180		<.001		<.001
No	76 (39.58)	8.97 ± 1.86		41.71 ± 4.92		49.09 ± 6.22	
Yes	116 (60.42)	9.38 ± 2.15		44.68 ± 5.51		53.56 ± 7.69	

**Table 3:** Distribution of answers for knowledge, attitude, and perception questions.

Statement			Correct	Incorrect	Unclear
1. The standard treatment for hematopoietic stem cell transplantation is to isolate patients in a laminar flow ward for about one month, during which safe and effective exercise should be performed to develop exercise habits			148 (77.08)	22 (11.46)	22 (11.46)
2. Exercise can improve muscle atrophy and joint stiffness, increase muscle strength, improve cardiovascular function, and alleviate fatigue			185 (96.35)	1 (0.52)	6 (3.13)
3. Taking corticosteroid drugs (such as prednisone) may lead to muscle weakness, muscle atrophy, and osteoporosis, therefore patients taking steroids should avoid exercise			68 (35.42)	51 (26.56)	73 (38.02)
4. Exercise helps increase appetite, improve sleep, regulate emotions, and reduce depression and anxiety			186 (96.88)	1 (0.52)	5 (2.6)
5. During hospitalization, bed rest is required, and high-intensity activities should be avoided, but it does not mean no exercise at all			51 (26.56)	127 (66.15)	14 (7.29)
6. Muscle strength is crucial because it is closely related to independence in daily activities			177 (92.19)	1 (0.52)	14 (7.29)
7. Muscle atrophy (muscle wasting) is one of the severe complications in hematopoietic stem cell transplant patients, affecting rehabilitation and prognosis			106 (55.21)	6 (3.13)	80 (41.67)
8. Patients’ low physical activity leads to a decrease in skeletal muscle mass, resulting in skeletal muscle atrophy and muscle loss			110 (57.29)	18 (9.38)	64 (33.33)
9. After transplantation, patients can engage in exercise if their physical condition permits, but they should proceed with caution and gradually increase the intensity			188 (97.92)	0 (0)	4 (2.08)
10. The principle of posttransplant exercise is to avoid exhaustion, starting from indoor activities and gradually increasing the intensity			179 (93.23)	7 (3.65)	6 (3.13)
11. Posttransplant activities can include walking, yoga, tai chi, and simple stretching exercises, preferably during less crowded times			183 (95.31)	3 (1.56)	6 (3.13)
12. To ensure safety, patients should engage in exercise when their platelet count is >10 × 10^9^/L, absolute neutrophil count is >0.5 × 10^9^/L, hemoglobin level is >80 g/L, and there are no severe infections or bleeding episodes, and no pathological fractures			130 (67.71)	15 (7.81)	47 (24.48)

Attitude	Strongly agree	Agree	Neutral	Disagree	Strongly disagree
1. During the transplantation period, the discomfort in my body makes me extremely miserable, and I have no desire to exercise at all	33 (17.19)	64 (33.33)	59 (30.73)	33 (17.19)	3 (1.56)
2. The discomfort during the transplantation period makes it difficult for me to intake nutrition, therefore, I am unable to exercise	25 (13.02)	58 (30.21)	56 (29.17)	49 (25.52)	4 (2.08)
3. Even if I know I can exercise, I have no desire to do anything during the transplantation period	24 (12.5)	53 (27.6)	48 (25)	60 (31.25)	7 (3.65)
4. Being confined to the ward makes me anxious, and I have no intention to exercise at all	17 (8.85)	39 (20.31)	55 (28.65)	71 (36.98)	10 (5.21)
5. If I hear that other patients also don’t want to exercise, I will be influenced and become resistant to exercise	15 (7.81)	19 (9.9)	43 (22.4)	102 (53.13)	13 (6.77)
6. The doctor talked to me about the benefits of exercise and advised me to exercise	62 (32.29)	108 (56.25)	20 (10.42)	2 (1.04)	0 (0)
7. The exercise regimen is relatively simple and easy	58 (30.21)	116 (60.42)	17 (8.85)	1 (0.52)	0 (0)
8. There is guidance, supervision, and encouragement from professionals in the field of exercise	51 (26.56)	108 (56.25)	26 (13.54)	7 (3.65)	0 (0)
9. Hearing other patients describe the benefits of exercise	52 (27.08)	114 (59.38)	23 (11.98)	3 (1.56)	0 (0)
10. Willpower is important, as it motivates me to continue exercise even if I feel physically uncomfortable, with the determination to resume after recovery	52 (27.08)	103 (53.65)	23 (11.98)	11 (5.73)	3 (1.56)
11. I focus on positive thoughts that exercise will make me better, so I will exercise actively	57 (29.69)	112 (58.33)	20 (10.42)	3 (1.56)	0 (0)
12. Exercise enables me to achieve my goals, so I will exercise actively	57 (29.69)	110 (57.29)	22 (11.46)	3 (1.56)	0 (0)
Perception					
The frequency at which you consider the following factors during the exercise process is (options ranked from high to low frequency):					

	Always	Often	Sometimes	Occasionally	Never
1.1. Quality of air	46 (23.96)	59 (30.73)	70 (36.46)	12 (6.25)	5 (2.6)
1.2. Availability of family members for accompanying	50 (26.04)	53 (27.6)	46 (23.96)	30 (15.63)	13 (6.77)
1.3. Weather changes and adjustments in clothing	63 (32.81)	82 (42.71)	42 (21.88)	4 (2.08)	1 (0.52)
1.4. Blood indicators	61 (31.77)	65 (33.85)	55 (28.65)	6 (3.13)	5 (2.6)
1.5. Presence of healthcare personnel accompanying	30 (15.63)	33 (17.19)	31 (16.15)	45 (23.44)	53 (27.6)
Below are the exercise types, please indicate your willingness to participate:					

	Very willing	Willing	Neutral	Unwilling	Very unwilling
2.1. Walking	91 (47.4)	89 (46.35)	12 (6.25)	0 (0)	0 (0)
2.2. Jogging/running	28 (14.58)	52 (27.08)	73 (38.02)	38 (19.79)	1 (0.52)
2.3. Climbing stairs	19 (9.9)	67 (34.9)	64 (33.33)	37 (19.27)	5 (2.6)
2.4. Cycling	28 (14.58)	70 (36.46)	55 (28.65)	34 (17.71)	5 (2.6)
3. Resistance training:					
3.1. Bodyweight exercises (e.g., sit-ups, squats)	17 (8.85)	48 (25)	73 (38.02)	52 (27.08)	2 (1.04)
3.2. Resistance bands	15 (7.81)	55 (28.65)	64 (33.33)	53 (27.6)	5 (2.6)
3.3. Dumbbells	16 (8.33)	39 (20.31)	64 (33.33)	67 (34.9)	6 (3.13)
3.4. Weight training equipment (common gym equipment such as Smith machine, leg press machine):	12 (6.25)	23 (11.98)	61 (31.77)	86 (44.79)	10 (5.21)
4. Respiratory training:					
4.1. Breathing technique training (e.g., pursed lip breathing, abdominal breathing)	42 (21.88)	91 (47.4)	40 (20.83)	19 (9.9)	0 (0)
4.2. Respiratory muscle training (e.g., resistance breathing, breathing exercises)	37 (19.27)	89 (46.35)	50 (26.04)	16 (8.33)	0 (0)

Comparison between groups showed the attitude total score (*P* < .001), positive attitude (*P* < .001), perception total score (*P* < .001), desire for exercise (*P* < .001), aerobic exercise (*P* < .001), resistance exercise (*P* < .001), and breathing training (*P* < .001) all exhibited substantial variations between patients previously inactive in exercise and those who were active. Furthermore, resistance to exercise demonstrated a notable difference (*P* = .018) between these groups. Notably, no significant variations were observed based on the duration after the transplantation procedure (Table 4).

**Table 4:** Comparison of answers according to the previous exercise habits and duration after the transplantation procedure.

Factor or statement	Patients		*P*	Patients		*P*
	Previously inactive in exercise (n = 76)	Previously active in exercise (n = 116)		Transplantation period within 1 year (n = 85)	Transplantation period over 1 year (n = 104)	
Knowledge	8.97 ± 1.86	9.38 ± 2.15	.180	9.49 ± 1.64	8.96 ± 2.32	.076
Attitude						
Total score	41.71 ± 4.92	44.68 ± 5.51	<.001	43.58 ± 5.39	43.44 ± 5.62	.868
Resistance to exercise (A1–A5)	13.74 ± 3.95	15.21 ± 4.31	.018	14.52 ± 3.86	14.75 ± 4.53	.708
Consistency in exercise (A6–A9)	16.24 ± 2.09	16.80 ± 2.28	.085	16.64 ± 2.53	16.51 ± 1.95	.700
Positive attitude (A10–A12)	11.74 ± 1.88	12.67 ± 1.83	<.001	12.42 ± 1.94	12.18 ± 1.88	.389
Perception						
Total score	49.09 ± 6.22	53.56 ± 7.69	<.001	51.79 ± 7.36	51.59 ± 7.51	.853
Factors to consider during exercise (P1.1–P1.5)	17.67 ± 3.29	17.91 ± 3.69	.642	17.72 ± 3.43	17.82 ± 3.64	.848
Desire for exercise (P2–P4)	31.42 ± 5.47	35.65 ± 6.28	<.001	34.07 ± 6.30	33.77 ± 6.33	.745
Aerobic exercise (P2.1–P2.4)	13.49 ± 2.56	15.16 ± 2.39	<.001	14.42 ± 2.45	14.52 ± 2.71	.801
Resistance exercise (P3.1–P3.4)	10.86 ± 2.87	12.59 ± 3.37	<.001	12.08 ± 3.18	11.71 ± 3.37	.441
Breathing training (P4.1–P4.2)	7.08 ± 1.42	7.91 ± 1.67	<.001	7.56 ± 1.74	7.54 ± 1.52	.912

**Figure 1.**
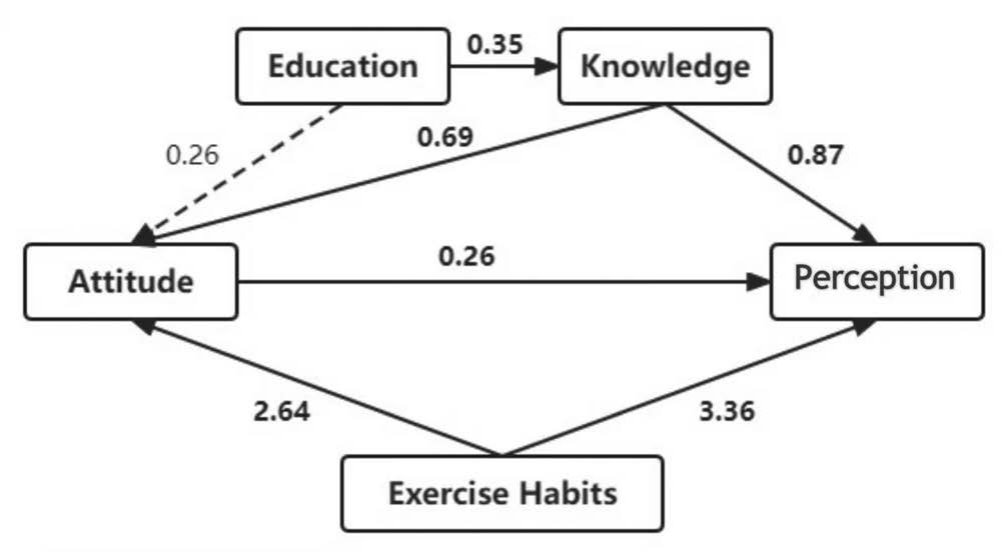
Results of the structural equation modeling, demonstrating the direct and indirect impacts of education level, exercise habits, attitude and knowledge on perception.

The SEM was used to test the hypothesis regarding the influence of knowledge on attitude and perception of exercises (Fig. 1). It was confirmed that perception was directly influenced by knowledge (β = 0.87, *P* < .001), attitude (β = 0.26, *P* = .007) and exercise habits (β = 3.36, *P* = .001), while attitude was influenced by knowledge (β = 0.69, *P* < .001) and exercise habits (β = 2.64, *P* < .001). In addition, education level had an indirect impact on perception (β = 0.44, *P* = .010) and attitude (β = 0.24, *P* = .019) through knowledge. Knowledge (β = 0.18, *P* = .029) and exercise habits (β = 0.68, *P* = .032) had an indirect impact on perception through attitude. In addition, positive correlation was found between knowledge and attitude (*R* = 0.293, *P* < .001) or perception (*R* = 0.316, *P* < .001) scores; attitude and perception scores (*R* = 0.317, *P* < .001) (Table 5).

**Table 5:** Correlation analysis.

	Knowledge	Attitude	Perception
Knowledge	1		
Attitude	0.293 (*P* < .001)	1	
Perception	0.316 (*P* < .001)	0.317 (*P* < .001)	1

## 4. Discussion

In this study, post-HSCT patients exhibited satisfactory knowledge, but moderate attitude and perception of exercises. Compared to patients actively engaged in exercises, previously inactive patients exhibited significant differences in knowledge scores and exercise perception. The SEM underscores education’s indirect impact on exercise perception. The study’s outcomes may offer practical insights for refining clinical practice in post-HSCT exercise rehabilitation. Addressing perceived barriers, enhancing knowledge, and promoting positive attitude can boost exercise engagement. Healthcare professionals should tailor education, target attitude, and personalize exercise plans to facilitate effective recovery post-HSCT.

To the best of our knowledge this is the first study to access KAP towards exercise in post-HSCT population. However, many barriers reported by patients are related to the previous condition that led to HSCT and chemotherapy treatment. Thus, based on the previous studies among cancer survivors it was expected that participation rates and adherence to rehabilitation exercises would be lower than in general population, despite good knowledge and understanding.^[23,24]^ Present study included post-HSCT patients who had previous exercise habit and those who did not exercise, expecting to compare the 2 groups in order to find differences in attitude perception that would explain different approach to practice. In addition, it was previously reported that with the longer time after discharge patients demonstrated worse knowledge of the disease and rehabilitation due to mainly undergoing educational interventions in hospital, during the main treatment.^[9,18]^ Obtained results partly confirmed above hypothesis, with attitude and perception scores found to be higher in patients who partook in exercises; however, knowledge scores were only slightly higher in those who received HSCT within 1 year, without statistically significant differences in any subdomains (all *P* > .05). It suggests that majority of post-HSCT patients still had adequate knowledge even 1 year after the procedure, while the exercising habit is influenced by a number of other factors.

Previous KAP studies undertaken among medical professionals and students reported sufficient knowledge and positive attitude, suggesting that healthcare specialists are ready to provide educational and moral support to the HSCT patients in the field of medical rehabilitation.^[16–18]^ On the other hand, recently published descriptive qualitative study by Yu et al^[19]^ discussed KAP towards exercises in 6 patients undergoing HSCT treatment; present study included 192 patients, who reported comparable physical and psychological barriers, such as fatigue, reduced appetite, decreased willpower, and anxiety. In addition, almost 15% of responders noted that negative attitude of other post-HSCT patients would influence their approach to exercise and about 7% noted that willpower is not as important as other factors. On the other hand, simplicity of the exercise regimen was among most important facilitators, with walking and breathing training being the most preferred types of exercise, while weight training was the least preferred type. It should be taken into account that HSCT procedure itself causes anxiety regarding the long-term side effects and concerns about the capability of the body to endure strain of complex exercises.^[16,17]^ Thus, guidelines for exercise and cancer published by American college of sports medicine,^[20]^ might be still actual for post-HSCT especially regarding individualized home-based exercise prescription until the full recovery of the immune system.

Although educational follow-up in post-HSCT patients contribute to improving compliance, knowledge alone not necessary translates into behaviors.^[15]^ In this study a positive correlation was confirmed of knowledge with attitude and perception, suggesting that educational interventions might be successful in the studied population; according to structural equation modeling perception of exercises was indirectly influenced by knowledge, education level and exercise habits via attitude. However, in addition to the simplicity of the regimen and knowledge obtained from doctors being important facilitators, this study uncovered that positive and negative attitude of other patients might also influence the perception to some degree. Study undertaken among Australian oncologists and general practitioners^[25]^ reported that despite having a positive attitude and willingness to recommend exercises to their cancer patients, the actual discussion of rehabilitation programs takes place less often. Although the majority of patients prefer to receive information in the hospital, the lack of encouragement from hematologist might lead to them turning to other sources.^[26]^ Moreover, after discharge patients are exposed to potentially unreliable information in the internet and television.^[27]^ Thus it is important to schedule meetings during the follow-up to discuss environmental factors that might influence negative opinions about exercise to improve medication compliance.

This study had several limitations. Although sample size was relatively big, some subs were under-represented. The mean age of patients was relatively young, thus their understanding and involvement in the treatment process were better, and the proportion of responders who previously partook in exercise was also higher. Despite previous study noted higher KAP scores and other specific features in women,^[17]^ this study did not divide patients according to gender. This study was based on the self-designed questionnaire and obtained results cannot be directly compared with other studies, limiting the generalizability. Moreover, difficulty level of the knowledge questions can directly affect the knowledge score, which is one of the serious limitations of KAP studies^[28]^; all knowledge questions in this study were carefully designed to address this limitation and underwent revisions based on feedback from hematology experts, with the following small-scale pilot study demonstrating acceptable reliability. And finally, additional bias is possible due to the retrospective nature of the study and social expectations of patients.

In conclusion, post-HSCT patients in this study had adequate knowledge, moderate attitude and perceptions of exercises, even 1 year after HSCT. Patients inactive in exercise exhibited significant differences in knowledge and exercise perception from active patients. Healthcare practitioners should customize education, focus on attitude, and individualize exercise plans to optimize post-HSCT recovery.

## Author contributions

**Conceptualization:** Na Han, Changqing Wu, Na Liu, Yu Deng.

**Data curation:** Na Han, Changqing Wu, Li Zhang, Yan Zhu.

**Formal analysis:** Na Liu, Yu Deng, Li Zhang, Yan Zhu.

**Investigation:** Na Han, Changqing Wu, Li Zhang, Yan Zhu.

**Methodology:** Na Han, Changqing Wu, Li Zhang, Yan Zhu.

**Writing – original draft:** Na Han, Changqing Wu, Li Zhang, Yan Zhu.

**Writing – review & editing:** Na Han, Changqing Wu, Na Liu, Yu Deng, Li Zhang, Yan Zhu.
